# Neonatal mortalities without detected risk factors before birth at Mtendeli hospital, Tanzania: a cross-sectional study

**DOI:** 10.11604/pamj.2024.47.15.39379

**Published:** 2024-01-15

**Authors:** Alen Kinyina, Sarah Mohamed Chamos, Hussein Moremi, Melkior Assenga, Mussa Bago, Renatus Mathias

**Affiliations:** 1 Faculty of Education, Health and Wellbeing, University of Wolverhampton, Wolverhampton, United Kingdom,; 2 Tanzania Red Cross Society, Mtendeli Hospital, Mtendeli, Tanzania,; 3 Muhimbili National Hospital, Dar es Salaam, Tanzania,; 4 Tanzania Health Promotion Support (THPS), Dar es Salaam, Tanzania; 5 Department of Public Health and Community Nursing, School of Nursing and Public Health, University of Dodoma, Dodoma, Tanzania,; 6 Tunduru District Council, Tunduru, Tanzania

**Keywords:** Neonates, mortality, pregnancy, antenatal, birth, Apgar score

## Abstract

**Introduction:**

neonatal mortality rate (NMR) is defined as the probability of dying during the first 28 days of life expressed per 1,000 live births. The death of neonates without risk factors at the end of pregnancy could be an indicator of sub-optimal quality care during labor and care of sick neonates. Therefore, this study aimed to determine the factors associated with neonatal deaths happening without detected risks during prenatal period.

**Methods:**

a cross-sectional study was conducted from 2017 to 2021.The recruited pregnant women were those who had a live, term, single-intrauterine pregnancy without detectable fetal abnormality at the time of starting labor. The data were collected through open data kit (ODK) forms that were customized in kobo tool in the tablets. The data analysis was performed using STATA statistical software. The factors associated with neonatal mortality were analyzed in a multiple logistic regression and considered significant if p < 0.05.

**Results:**

among the 4401 enrolled mothers, neonatal deaths were 361 (8.2%). The factors associated with death of neonates without risk factors during prenatal period were low Apgar score [AOR = 4.38: 95%CI (2.33-7.72)], male sex [AOR=2.25: 95%CI (1.12-3.81)], gestational age above 40 weeks [AOR=4.79: (2.50-7.61)] and assisted vaginal delivery [AOR = 2.55: 95%CI (1.12-4.96)].

**Conclusion:**

the increased number of neonatal deaths are associated with sex of neonates, low Apgar score, post maturity and assisted vaginal delivery. The hospital-based studies should be done to address the preventable neonatal deaths with no detected risk factors before birth.

## Introduction

Globally, there is significant progress in reduction of neonatal mortality. NMR has decreased from 37 to 18 per 1,000 live births in 1990 and 2018 respectively [1]. However, NMR contributed to the increase of under-five mortality rate from 40% to 47% between 1990 and 2018 [1]. Worldwide, 75% of 2.5 million neonatal deaths that were reported in 2019, 35% was due to prematurity, 24% contributed by birth asphyxia, and neonatal sepsis contributed 15% [2]. Sub-saharan African countries have the high neonatal mortality rate as estimated to be 28 per 1,000 live births in 2018 [3]. The majority of these neonatal mortalities could be prevented through the improvement of adequate skilled healthcare workers attending deliveries, provision of quality of antenatal care (ANC), postnatal care and the care of sick neonates [4]. The reduction of at least the rate of 4.3% per year is recommended for attaining the NMR of < 12 per 1,000 live births proposed in the sustainable development goal (SDG) by 2030 [5].

Tanzania is experiencing a slow reduction of neonatal mortality rate compared to the under-five mortality. NMR decreased from 26 to 25 per 1,000 live births between 2010 and 2015 [6]. The leading causes of neonatal mortality in Tanzania are birth asphyxia (33%), premature births (28%), and infections (24%) [7]. The achievements of reducing the under-five mortalities depend greatly on the efforts to address the neonatal mortalities as they are contributing to 39% of under-five mortality [5]. So far various interventions have been undertaken to decrease the NMR including quality improvement of labor monitoring, which is a key in prevention of birth asphyxia, the major cause of neonatal mortality in the country [8]. The SARA report shows that Tanzania has increased the number of health facilities that are capable of using partograph in management of labor to 77% [7]. Furthermore, Tanzania has increased the number of skilled attendants by improving their skills through on job training and reshuffling of staff in the major consultant hospitals [9]. The country has also increased facilities that provide the essential newborn care services [10]. Despite of these interventions, studies have reported suboptimal quality care which results in poor birth outcomes [8,9]. Fresh stillbirths (FSB) are increasing over time in the country with an estimate of around 47,000 deaths annually [10]. This corresponds to a rate of 25.9 per 1000 births: this is the ninth highest rate globally [10].

Most of the neonatal death surveillance and estimation of mortality rate are conducted through cross-sectional, case-control or prospective studies by reviewing the previous patients´ files usually at regional or national level [8]. These studies are conducted in a short period utilizing the information from a small sample of respondents [11]. The review of the past patients´ files has a lot of limitations including underreporting of data, inaccuracy of information and reviews are not frequently conducted [8]. In addition, none of these studies address the neonatal deaths without risk factors previously detected during prenatal period. There is a need to address the neonatal deaths happening without any detected risk factors before starting labor in order to ascertain the preventable deaths during labor and postnatal period. Therefore, this study was conducted as a cross-sectional hospital-based study to determine the factors associated with neonatal deaths occurring without predetermined risks during prenatal period. The findings will serve as a starting point of all lower-level hospitals in conducting retrospective assessment of neonatal deaths to track the progress of achievements towards the SDG on improvement of child health that aim to end the preventable neonatal deaths.

## Methods

**Study setting** : this study was carried out at the sexual and reproductive health department of Mtendeli hospital. Mtendeli is a secondary level hospital which attends the Burundian refugees and Tanzanian patients from and out of Kigoma region. This big hospital attends around 2500 women with an estimate of 1800 deliveries and 103 neonatal deaths each year.

**Data collection**: the study utilized a cross-sectional study design carried out for 5 years from 2017 to 2021. The enrollment of the study participants was done by the trained health personnel who were able to conduct the obstetric ultrasound and identify clearly all eligibility criteria. The data were collected by the nurses/midwives who were trained on the use of Open Data Kit (ODK). The data collection forms were customized into the kobo collect tool and collection of data were done by using smartphone or tablets. The research coordinator checked the accuracy and completeness of the information in the database weekly.

**Inclusion criteria**: to be included in this study, the pregnant woman had to be physically and mentally healthy with a single-intrauterine pregnancy without detectable fetal abnormality. She had to have undergone an early obstetric ultrasound during admission to confirm a term and live intrauterine fetus with longitudinal lie. The term of the pregnancy was determined by the method of gestation age (GA) evaluation proposed by Eregie model [12]. The live intrauterine fetal status was esteemed normal if the fetal heart rate (FHR) was between 110 and 160 beats per minute [3]. The women must also have been assisted in delivery by the skilled health personnel after starting labor with a live fetus. Each selected participant was recorded in designed Open Data Kit (ODK) form and the information were stored safely in the database for future review and analysis.

**Exclusion criteria**: a woman was not included in this study if she had multiple pregnancy, is not physically or mentally healthy, is admitted in labor without obstetric ultrasound or those who were admitted in the second stage of labor. Furthermore, women with detected intrauterine fetal malformations were excluded in this study. All intrauterine deaths or non-reassurance fetal status at the time of starting labor were not included.

**Variables of interest**: the researcher collected the anthropometric variables (age, weight, height) and obstetric data of pregnant women (number of parity, mode of delivery and GA at delivery) as well as parameters of newborns at birth (sex, birth weight (BW) and Apgar score at the first and fifth minute). Newborns´ BW were divided into three groups (< 2500g; 2500-2900g; ≥4000g). The Apgar score was considered low (<7 score) and Good (≥7 score). Age was grouped as: < 20 years old; 20-34 years old and ≥35 years, Parity categorized as (primipara giving birth the first time; multiparous as 2-5 parities; grand multiparous for parity of ≥6), and GA (37-38 weeks; week 39-40 weeks; 41-42 weeks). The women also were classified according to the number of antenatal care (ANC) (<4 ANC visits and ≥4 ANC visits). Vital outcomes (alive or death) were recorded at birth and/or in the early neonatal period.

**Data analysis**: data analysis was done by using STATA software version 14 for windows (StataCorp LP,College station,Tx,USA). The results were presented as mean ± standard deviation or in absolute and relative frequencies (in percentage). Demographic factors, obstetrical and neonatal factors significantly associated with neonatal mortality were analyzed in a multiple logistic regression analysis. The result was considered significant if p < 0.05.

**Ethical considerations**: the study respected the rules of anonymity and confidentiality, the protocol of this study was approved by the ethics committee of the district health management team. All participants signed the written consent forms after being informed the aim of this study.

## Results

**Figure 1:**
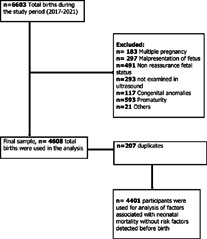
flow diagram for included and excluded samples at Mtendeli hospital

**Total admissions and eligible participants of the study**: during the study period there was a total of 6603 admissions in the hospital for the reasons related to labour and delivery (This sample included women with one or more deliveries during the study period). All women were screened for eligibility criteria and the study protocol to give a final sample of 4401 who were included in the final analysis (Figure 1).

**Characteristics of the study participants**: during the 5 years of conducting this study, we enrolled pregnant women who gave birth to 4401 newborns, including 4040 (91.8%) live newborns and 361(8.2%) early neonatal deaths. Table 1 presents all newborns in categories according to their birth variables and maternal characteristics. The number of newborns included 2117 girls (48.1%) and 2284 boys (51.9%). The mean BW was 3101 ± 329 g. The mean (± standard deviation) of age, weight and height of the mothers was 27.9 ± 4.6 years, 67.1 ± 8.7 kg and 161 ± 4 cm respectively. The mean GA was 38.3 ± 1.2 Weeks. The proportion of neonatal deaths was higher (p = <0.001) among boys (8.8%) than girls (8. 1%). The birth weight (BW) of neonatal death (3101 ± 407 g) was similar (p = 0.012) to that of live newborns (3072 ± 409 g), but the Apgar score in these two groups was significantly different (p=< 0.001) (Table 1).

**Table 1:** general characteristics of the study participants

Variables	Alive(n=4040)	Neonatal mortality (n=361)	Total (n =4401)	P-Value
**Sex**				
Female	1946 (91.9%)	171(8.1%)	2117(48.1%)	< 0.001
Male	2083 (91.2%)	201 (8.8%)	2284 (51.9%)	
Birth Weight	3072± 409	3101 ± 407	3101± 329	0.012
**Apgar score** (5 min)				< 0.001
Gestation Age (GA)	37±.11	39±2.1	38.3 ± 1.2	< 0.001
Maternal Age (Years)	27.8 ± 4.7	27.9 ± 4.5	27.9 ± 4.6	0.033
Maternal weight (kg)	66.3 ± 8.2	67.1 ±8.5	67.1 ± 8.7	0.226
Maternal height (cm)	159 ± 5	161±4	161 ± 4	0.951
Parity	3.6 ± 2.1	3.8 ± 2.3	3.5 ± 1.4	0.025
ANC visits	4.2±0.8	4.6± 1.9	4.4 ± 1.1	< 0.001

**Figure 2:**
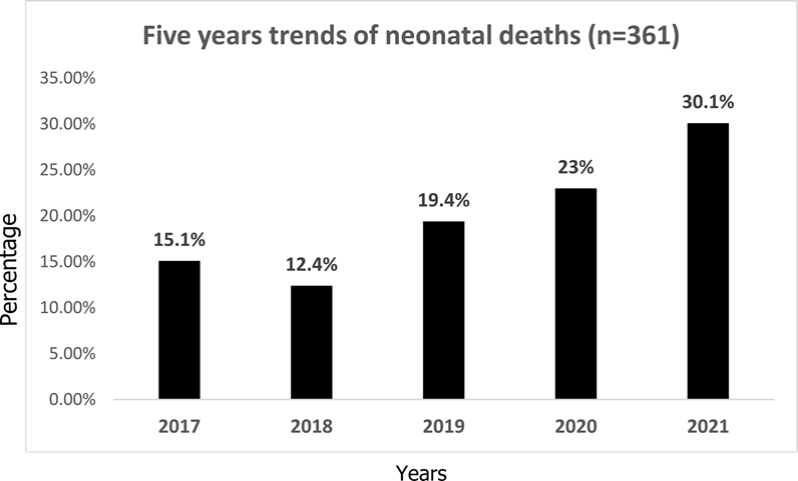
trends of neonatal death from 2017 to 2021 at Mtendeli hospital

**Trends of neonatal deaths at Mtendeli hospital**: in five years from 2017 to 2021 the hospital experienced the increase of neonatal deaths from 15.1% in 2017 to 30.1%. The proportion of death has been increasing consecutively for three years between 2019 -2021 (**Figure 2**).

**Obstetrics and neonatal characteristics of the participants**: the majority of mothers were aged 20-34 years (82.0%), were multiparous (66.1%), followed <4 ANC (64.9%) and 95% delivered through spontaneous vagina delivery (SVD). About the variables of the newborn, the majority of newborns had a BW of 2500-3999g (88.5%) and a “Good” Apgar score (83.8%). The NMR increased significantly (p <0.001) with low Apgar score by 40.3%. The rate of neonatal death was not statistically different in various maternal age categories (p = 0.285) but increased (p <0.001) in the group of mothers who followed ≥4 ANC visits (13.2%) compared to <4 ANC visits (5.5%) and varied from 18.3% in the case of assisted vagina delivery (SVD) to 7.9% for SVD and 9.1% cesarean section, (p <0.001). The neonatal mortality was statistical significance according to the increased GA (p <0.001) (Table 2).

**Table 2:** univariate analysis of obstetric and neonatal factors associated with neonatal mortality

Variables	Alive n(%)	Neonatal mortality n(%)	Total number of deliveries n(%)	X2	P-Value
**Birth weight (BW)**					
< 2500	311(95.1)	16(4.9)	327(7.4)		
2500 - 3999	3556 (91.3)	338 (8.7)	3894 (88.5)	4.241	0.153
≥ 4000	173(96.6)	7(4.0)	180 (4.1)		
**Apgar score**					
Good	3614(98)	73(2.0)	3687(83.8)	11.229	< 0.001
Low	426 (59.7)	288(40.3)	714(16.2)		
**Maternal Age (Years)**					
< 20	132(94.3)	8(5.7)	140(3.2)		
20 - 34	3302(91.4)	309 (8.6)	3611(82.0)	3.226	0.285
≥ 35	606 (93.2)	44 (6.8)	650 (14.8)		
**Parity**					
Primipara	780(92.0)	68(8.0)	848 (19.3)		
Multipara	2694(92.6)	215(7.4)	2909 (66.1)	3.117	0.219
Grand Multipara	566 (87.9)	78 (12.1)	644 (14.6)		
**ANC**					
ANC visitï¿½ < 4	2697(94.5)	156 (5.5)	2853 (64.9)	10.748	< 0.001
ANC visit ≥ 4	1343(86.8)	205(13.2)	1548(35.1)		
**Mode of delivery**					
SVD	3852 (92.1)	330 (7.9)	4182(95.0)		
Assisted Vaginal delivery	98 (81.7)	22 (18.3)	120(2.7)	16.582	< 0.001
Caesarean section	90 (90.9)	26 (22.6)	89 (77.4)	115 (2.6)	
9 (9.1) 99 (2.3) **GA at birth (weeks)** 37-38 2911 (94.6) 165 (5.4) 3076 (69.9) 39-40 1103(91.2) 107 (8.8) 1210 (27.5) 14.613 < 0.001 41-42					

**Factors associated with neonatal mortality among 4401 newborns in the multivariate analysis**: after controlling of the potential confounders, the odds of neonatal mortality among male neonates was 2.2 times higher compared to female [AOR=2.25: 95%CI (1.12-3.81)]. The odds of neonatal mortality among neonates who had a low Apgar score was 4.4 times higher compared to neonates who had good Apgar score [AOR = 4.38: 95%CI (2.33-7.72)]. Neonates from women with 4 or more ANC visits during prenatal period have 2.1 times higher odds of neonatal death compared to neonates from women who agreed to less more than ANC visits during pregnancy [AOR = 2.11: 95%CI (1.18-4.84)]. Furthermore, neonates who were born at 41-42 weeks had 4.8 times greater odds of neonatal mortality compared to neonates from women who delivered at GA of 37-38 weeks [AOR=4.79: (2.50-7.61)]. The odds of neonatal mortality among neonates born through assisted vaginal delivery was 2.6 times higher compared to neonates born through SVD [AOR = 2.55: 95%CI (1.12-4.96)] (Table 3).

**Table 3:** factors associated with neonatal mortality among 4401 newborns in the multivariate analysis

Variables	Neonatal death		Crude OR (95% CI)	Adjusted OR (95% CI)
	**No**	**Yes**		
**Sex**				
Female	1946 (91.9%)	171 (8.1%)	1	1
Male	2083 (91.2%)	201 (8.8%)	2.16(1.22-3.38)	2.25(1.12-3.81) *
**Apgar score**				
Good	3614 (98)	73(2.0)	1	1
Low	426 (59.7)	288(40.3)	3.71 (2.37–5.074)	4.38(2.33-7.72) **
**ANC**				
ANC visit <4	2697 (94.5)	156 (5.5)	1	1
ANC visit ≥4	1343(86.8)	205 (13.2)	2.44 (1.24–3.93) **	2.11 (1.18–4.84) *
**GA at birth (weeks)**				
37-38	2911 (94.6)	165 (5.4)	1	1
39-40	1103(91.2)	107 (8.8)	1.09(1.11-2.35)	1.31(0.65-3.16)
41-42	26 (22.6)	89 (77.4)	5.47(3.28-8.62)	4.79(2.50-7.61) **
**Mode of delivery**				
SVD	3852 (92.1)	330 (7.9)	1	1
Assisted vaginal deliver	98 (81.7)	22 (18.3)	2.42(1.33-3.85)	2.55(1.12-4.96) **
Caesarean section	90 (90.9)	9 (9.1)	1.54(1.19-3.71)	1.61(0.73-2.97)

* p<0.05. **p<0.001

## Discussion

This study assessed the maternal and fetal factors associated with neonatal deaths with no risk factors detected before birth among 4401 neonates delivered in a secondary level hospital where prenatal consultations as well as deliveries are provided by skilled health personnel. These births were from pregnant women apparently in good health, with term intrauterine pregnancy, with a single fetus, alive and without detectable abnormalities at the time of starting labor. The findings of this study indicate that, the deaths without risk factors before birth were 361(8.2%) newborns, i.e. 8.1% of girls and 8.8% of boys. In univariate analysis, the BW, the Apgar score, the demographic characteristics (age and weight) were associated with neonatal mortality. The obstetric characteristics of the pregnant women (number of ANC visits, parity, mode of delivery and GA) were also found to be associated with NMR. In the multivariate logistic analysis, newborn sex, Apgar score, number of ANC, mode of delivery and GA found as factors significantly associated with NMR. The analysis of factors influencing neonatal deaths have been the subject of discussions in many studies of maternal and newborn health [13].

However, the deaths happening and their associated factors at the end of pregnancy remains particularly difficult in many discussions due to high percentage of deaths happening with unknown cause as evidenced by the proportion of 8.2% of deaths without risk factors before birth in the present study (Table 1). However, this proportion may be low, following the strict selection criteria of pregnant women who participated in this study, and underestimating the stillbirths that happened to women who did not participate in the study. This may explain why it appears lower than the NMR rates of 10.9% and 24.3% reported respectively by Udo *et al*. in Nigeria [14] and Moyen *et al*. in sub-Saharan countries [15]. However, this rate is much higher compared to the findings reported in the developed countries [16]. This may reflect the inefficiency of health care services in our context. The higher NMR in boys compared to girls in this study corroborated with findings in other studies [8,13,14]. The reasons for such higher NMR without risk factors in boys are not clear. Many authors [14,17,18] indicate that, from the fetal period to all ages of childhood, boys have a higher mortality than girls even when the care and value placed by society on both sexes are similar. Intrauterine fetal death is also higher in male fetuses, and girls grow more quickly than boys [18].

While BW does not appear to have affected NMR in this study, so did the Apgar score. The low Apgar score by the fifth minute has been associated with increased NMR, similar to other literature findings [8,13]. However, in this review, we did not specifically investigate factors that may have resulted in low Apgar score in term newborns; such factors may have been triggered by the mode of delivery. While the age, weight, and height of the mother showed no association with NMR without risk factors during prenatal, obstetric variables, on the other hand, can influence NMR. In this study there were no significant differences of proportion of death among the newborns born to primipara mothers and multiparas. However, these obstetric risks can contribute to both fetal and maternal death [9]. On the other hand, based on the logistic analysis, the proportion of death in the present study was higher in newborns whose mothers had followed at least four ANC visits. The World Health Organization recommends an average of four antenatal visits during pregnancy [19]. The mean frequency of 4.4 ANC followed by our pregnant women complies with this recommendation.

Although the number of ANC among mothers with neonatal mortality exceeds that of mothers with live neonates, taking into account other factors, clearly shows a substantial decrease in the proportion of deaths in the group of pregnant women having followed 4 or more ANC. Such findings suggest that close monitoring of pregnant women will ultimately have been more beneficial for the outcome of the pregnancy. Our results agree with those of Hug *et al*. [16] who reported the fact that, the more the pregnant is monitored at ANC, the lower she is at risk of neonatal death for her pregnancy. However, some pregnant women tend to have more ANC visits than others due to prolonged duration of pregnancy. The mean GA at delivery was 38.2 ± 1.1 weeks. Not only was this GA greater in mothers with neonatal death, but the likelihood of neonatal death also increases as the GA increased above 40 weeks. Such a significant association of neonatal deaths with gestation age corroborates the data in other studies [8,10]. The higher mortality observed as a result of the increased GA indicates the greater vulnerability usually reported in the case of GA of 42 weeks defined as post-maturity [2,20].

This study did not specifically explore the characteristics of post-maturity among pregnant women. In low level settings the detection of post-maturity depends on if a woman is sure of the date of conception. Post-term pregnancy results to the increase of depletion of the placenta and oligohydramnios which can cause compression during labor and fetal hypoxia which consequently lead to fetal death [20]. In addition, changes in fetal heart rate are much more common in post-term compared to term newborns. Fetal ultrasound is a fundamental in the diagnosis of post maturity by studying placental grading, fetal growth and the amount of amniotic fluid [20]. The most characteristic histological abnormalities of the post-term placenta are the presence of intense calcium deposition [21], degeneration and a decrease in the perfusion of the villi responsible for the decrease in the functional value of the placenta and the 'possible alteration of maternal-fetal exchanges [21]. Certain limitations should be considered in the interpretation of the neonatal mortalities without risk factors in this study. The strict selection of pregnant women included in the analysis eliminated many known causes of neonatal death. Practice of autopsies on deceased newborns which are not done in our hospital would probably have revealed etiological factors that we were unable to identify. On the other hand, the inclusion criteria of only pregnant women with live fetuses at time of starting labor probably reduced the record of stillbirths (fetus died before starting labor).

## Conclusion

There is unacceptable high number of neonatal deaths without risk factors before birth. The majority of these mortalities are linked to sex of neonates, low Apgar score, post maturity and assisted vaginal delivery. These indicates that many neonatal deaths are happening at the end of pregnancy without antenatal risks. However, the findings of this study call the attention of the government and other stakeholders to address preventable deaths through conducting the hospital based cross-sectional studies, provision of the quality antenatal, intrapartum care and care of sick neonates.

### What is known about this topic

- *There is high neonatal mortality rate in sub-Saharan African countries*;
- *The majority of neonatal deaths happen in the hospitals in the first seven days of life*.

### What this study adds

- *The contributing factors of neonatal mortalities without detected risk factors before birth*;
- *Addressed factors linked to NMR indicate the sub-optimal quality care during labour and care of sick neonates*;
- *Hospital based cross-sectional study is a good design which can be used to track the trends of NMR in the lower-level settings*.
